# Active surveillance for safety monitoring of seasonal influenza vaccines in Italy, 2015/2016 season

**DOI:** 10.1186/s12889-018-6260-5

**Published:** 2018-12-22

**Authors:** Stefania Spila Alegiani, Valeria Alfonsi, Eva Charlotte Appelgren, Lorenza Ferrara, Tolinda Gallo, Cristiano Alicino, Maria Grazia Pascucci, Silvia Aquilani, Antonietta Spadea, Silvio Tafuri, Caterina Rizzo

**Affiliations:** 1 National Center for Drug Research and Evaluation, Istituto Superiore di Sanità, Rome, Italy; 2 Department of Infectious Disease, Istituto Superiore di Sanità - Italian National Institute of Health, Viale Regina Elena 299, 00161 Rome, Italy; 3 SeREMI, Local Health Unit of Alessandria-Piedmont Region, Alessandria, Italy; 4 Department of Prevention, Local Health Unit 4 Medio Friuli, Udine, Italy; 5 Department of Health Sciences, University of Genoa, Genoa, Italy; 6 Directorate General for Health and Social Policy - Emilia-Romagna Region, Bologna, Italy; 7 Viterbo Local Health Unit, Lazio Region, Viterbo, Italy; 8 RM/1 Local Health Unit, Lazio Region, Rome, Italy; 9 Department of Biomedical Science and Human Oncology, University of Bari, Bari, Italy; 10 Unit of Innovation and Clinical Pathways, Bambino Gesù Children’s Hospital, Rome, Italy

**Keywords:** Influenza, Vaccines, Safety, Surveillance

## Abstract

**Background:**

Surveillance for adverse events following immunization is an important component of any national immunization programme because it is critical to assessing the safety of vaccines and to detecting potentially rare and severe adverse events and responding in a timely manner. We conducted an enhanced active surveillance aimed at assessing the safety of flu vaccines in the 2015–2016 season in Italy. The study was targeted to the population groups for which the seasonal vaccine is recommended in Italy.

**Methods:**

During the study period, a total of 3213 individuals receiving seasonal influenza vaccination were registered on the web-based platform. Any adverse events experienced after 7 days from vaccination by individuals aged six months or more were collected through a telephone interview or by a web-based self-administered questionnaire. All individuals experiencing at least one event during the 7 days of follow-up were contacted for follow-up to 60 days.

**Results:**

Overall, 854 events were reported: 845 events (26%) after administration of the first dose and 9 (12%) after the second dose. The majority of adverse events reported after 7 days from the first dose were of little clinical importance, and most involved local symptoms.

**Conclusion:**

Our data, even though the number of vaccinated individuals was smaller than expected, is consistent with the safety of influenza vaccines in Italy during the 2015–2016 season regarding the most common adverse events. Further efforts are needed to obtain sufficient power to study rarer adverse events. Active monitoring and systematic studies to test generated signals and hypotheses are crucial to intensify awareness among the public and professionals with regard to the safety of vaccines.

**Electronic supplementary material:**

The online version of this article (10.1186/s12889-018-6260-5) contains supplementary material, which is available to authorized users.

## Background

Influenza is a major public health problem; a conservative estimate suggests that the illness infects every third child and every tenth adult, annually 60 million of the 500 million inhabitants of the European Union. It is related to 50 million episodes of mild clinical disease, 150 thousand hospital admissions and 15 to 40 thousand deaths annually [1–5].

Vaccines are the cornerstone of prevention for influenza and its consequences [6]. However, in the last two decades, controversies regarding the effectiveness and safety of influenza vaccines have triggered extensive epidemiological research and networking, an effort that is further strengthened by the need for pandemic preparedness [7].

In Italy, seasonal influenza vaccination is recommended and provided free of charge to the elderly (≥ 65 years) and to people aged 6 months to 64 years with comorbidities that increase the risk of influenza complications and to those in other categories (e.g., pregnant women in the second and third trimester, health care workers, etc.) [8]. Vaccine safety surveillance is passively undertaken by the spontaneous reporting system of the Italian national pharmacovigilance system, through the collection and analysis of adverse events following immunization (AEFI) based on spontaneous reports from doctors, health professionals and patients. Through these reports may emerge signs that suggest a need for supplementary investigation to deny or confirm issues and to quantify them in terms of risk through ad hoc pharmacoepidemiology studies.

Therefore, pharmacovigilance is mostly inadequate in its ability to early detect events; a renewed approach is essential and necessary to quickly and effectively track vaccine-related AEFI and to provide reliable, near real-time data and denominators on numbers of those vaccinated. Additionally, such types of studies also allow an assessment of the safety profile of the vaccine by product and brand, unlike pharmacovigilance, which is lacking in denominators and suffers from under reporting. Accordingly, the European Medicines Agency (EMA) has released regulatory guidance on enhanced safety surveillance for seasonal influenza vaccines in the EU [9].

In this regard, pharmacoepidemiology studies conducted in different populations to evaluate influenza vaccine safety show that local side effects, such as swelling, redness and pain at the injection site, which generally are mild and rarely interfere with daily activities, are common, occurring in more than 10% of recipients. Fever, tiredness and myalgia also occur commonly (1–10%). These adverse events may be more pronounced in children [10–13].

The Italian National Institute of Health (ISS) has the experience and organization necessary to conduct influenza vaccine effectiveness and safety studies at the national level through the involvement of local health units (LHU), general practitioners (GPs) and paediatricians, using ad hoc tools [10, 14–16].

This paper presents findings from a study conducted during the 2015–2016 season in Italy, targeted to the population group for which the influenza vaccine is recommended and aimed at implementing enhanced surveillance to evaluate influenza vaccine safety.

## Methods

### Study setting and population

An invitation letter was sent through Italian interregional prevention coordination to vaccine centres (GPs, paediatricians, hospitals and vaccine services of LHU) in 6 selected Italian Regions (Piedmont, Liguria, Friuli Venezia-Giulia, Emilia Romagna, Lazio, Apulia) for involvement in the study.

All individuals aged 6 months or more receiving the seasonal influenza vaccine in vaccine centres who spontaneously approached the vaccine centres were asked to participate. Eligible participants were those who provided signed written informed consent (parental consent was obtained for individuals aged < 18 years) and who agreed to be interviewed by telephone or to fill in a web-based questionnaire 7 days after vaccination.

### Organization of the study

Information on vaccinated individuals as well as regarding vaccine brand, date of vaccination, medical conditions, and self-reported history of allergy and influenza vaccination in the previous season were collected, during the medical pre-vaccination assessment, through a case report form (CRF, Additional file 1) by vaccine centres. Then, each recruited individual was given a diary to record any event or reaction and healthcare consultation that occurred within 7 days of vaccination, including visit to a GP, access to an emergency department or hospitalization.

After 7 days (follow-up to 7 days according to the EMA guidelines [9]), all parents (in the case of subjects aged less than 18 years old) and vaccinated individuals were asked to communicate any AEFIs reported in their diary during a telephone interview. In one region (Emilia Romagna) it was also proposed that participants directly enter these data on a web-based platform. All information was collected through a standardized questionnaire that included a list of events in a drop-down menu (Y/N) and a free text option for other responses not included in the menu.

AEFIs included local events, such as redness, pain, and indurations, and systemic events, such as fever (above 37.5 °C), chills, myalgia, arthralgia, general weakness, headache, syncope, nausea, and vomiting. Serious adverse events (SAE) were categorized according to predefined criteria, which included any unexpected medical event that resulted in death, was life-threatening or required hospitalization [17]. We also included seizures requiring medical attendance (emergency department visits and/or hospitalization) as medically important events.

All individuals experiencing at least one event during the 7 days of follow-up were contacted by telephone 60 days after vaccination to monitor the course of the events (follow-up to 60 days).

Data for the study were compiled from mid October 2015 through March 2016.

Data were entered into a web-based database, which was protected in terms of security. The enrolment and input of information from the vaccinated individuals was monitored through the web-based platform to highlight in real time possible errors or data quality issues (i.e., regarding date of vaccination, name of vaccine, outcome of the follow up).

Suspected adverse reactions observed during follow-up of the vaccinated individuals were reported to the National Network of Pharmacovigilance by vaccine centres using a standard form printed directly from the web-based platform.

The study was approved by the Ethical Committee of the National Institute of Health.

### Statistical analysis

The frequency of reported AEFIs was computed as the ratio between vaccinated individuals who reported at least one event and the total number of vaccinated individuals.

The association between the characteristics of participants and the type of vaccine and the occurrence of at least one event during the 7 days of follow-up was analysed through a Chi-square test for categorical variables. The multivariate logistic regression model was performed taking into account all potential confounding factors available from the CRF. In the final model, we included all variables with *p* < 0.05 in the univariate analysis, excluding influenza vaccination in the campaign 2014–2015 because of the large amount of missing of data. SPSS software (IBM SPSS Statistics, version 22) was used for the statistical analyses.

## Results

**Fig. 1:**
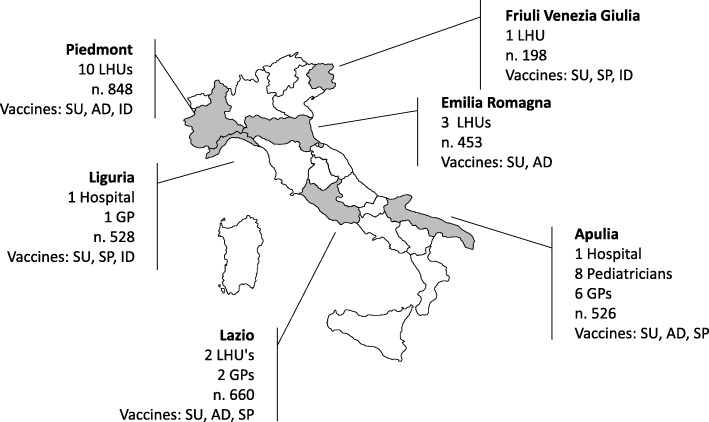
Number of centres, number of vaccinated subjects and type of vaccines by region. *LHU: local health unit; GP: general practitioner; SU: subunit; AD: adjuvanted; SP: split; ID: intradermal*

A total of 9 GPs, 8 paediatricians, 2 hospitals and 13 vaccine services of LHUs, distributed in 6 Italian Regions participated in the study (Fig. 1).

Seven different available commercial products were included in the study: two subunit vaccines, three split vaccines, one adjuvanted vaccine and one intradermal vaccine. Only subunit vaccines were used by all participating regions; split vaccines were used in 4 regions and adjuvanted and intradermal vaccines in 3 regions.

**Fig. 2:**
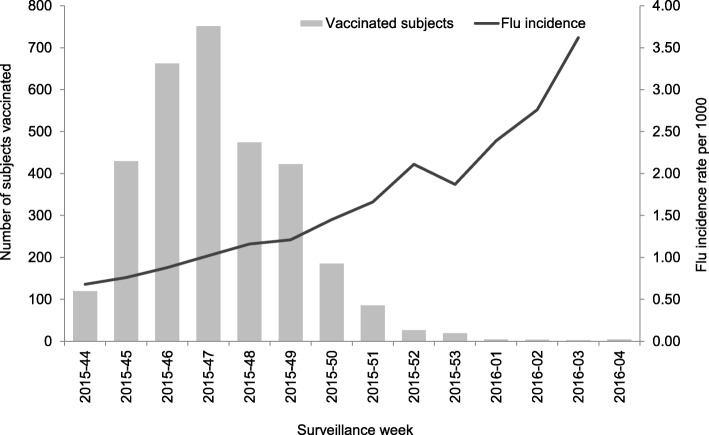
Number of vaccinated subjects and flu incidence by week of vaccination, 2015–2016 season

During the study period, a total of 3213 individuals receiving seasonal influenza vaccination, who provided written informed consent, were registered into the web-based platform, 73 of whom received two doses of vaccine (Fig. 2).

The median age of the study population was 57 years (range 8 months to 100 years), 20% were paediatric patients aged 6 months-14 years, 4% were aged 15–25 years and 38% were more than 65 years old; the male-to-female ratio was 1 (1614 males and 1599 females). Half of the participants received a subunit vaccine (*n* = 1636), 21% (*n* = 660) a split vaccine, 16% (*n* = 526) an adjuvanted vaccine and 12% (*n* = 394) an intradermal vaccine.

The majority of subjects were high-risk individuals (70%); this included those who were older, pregnant women and those with chronic health conditions who are the target of the influenza vaccination programme in Italy [8]. Approximately 40% of these individuals had an underlying medical condition, mainly cardiovascular and respiratory in nature. A total of 1958 participants (61%) had also been vaccinated against influenza in the previous season (2014–2015).

The number of telephone interviews performed for the 7-day follow-up was 3152, and 49 vaccinated individuals directly entered data on the web-based platform; therefore, the follow-up to assess any event within 7 days of vaccination was completed for 97% (*n* = 3201) of vaccinated individuals. All the 49 vaccinated participants entering data via web were recruited from the Emilia Romagna region, the only one that decided to apply the web-based approach, and they were younger (median age 53 vs 58 in the study population) and mainly vaccinated with the subunit vaccine (86%) as was the vaccine available for this age group.

Overall, 854 AEFIs were reported: 845 events (26%) after administration of one dose and 9 (12%) after the two doses. The proportion of AEFIs reported varied between 21 and 22% of the split and adjuvanted vaccines to 27% of the subunit vaccines and 38% of the intradermal vaccine (Table 1). The majority of AEFIs reported 7 days from vaccination were of little clinical importance (Table 2), except for two SAE, which occurred after the administration of split vaccines. These involved two elderly individuals (a male age 76 and a female age 87) with severe chronic underlying conditions who died three days (during a hospitalization) and ten days after the vaccination, respectively. No further hospitalizations were reported but one ED visit for conjunctivitis was noted. The most frequent AEFIs reported were local symptoms (pain, *n* = 446; redness, *n* = 222; swelling, *n* = 209; and induration, *n* = 174), followed by malaise (*n* = 122), fever ≥37.5 °C (*n* = 75), headache (*n* = 72) and arthralgia (*n* = 42), with no significant differences for type of vaccines administered (Table 2).

**Table 1:** Vaccinated individuals with at least one event within 7 days of vaccination by geographical area, type of vaccine and characteristics

	Number of subjects vaccinated^a^		
	Total	With any event (%)	OR (95% CI)
Geographical area			
Nord-west (Piedmont, Liguria)	1376	352 (25.6)	*1*
Nord-east (Friuli Venezia Giulia, Emilia Romagna)	651	203 (31.2)	1.32 (1.07–1.62)
Centre-South (Lazio, Apulia)	1186	290 (24.5)	0.94 (0.79–1.13)
Type of vaccines^b^			
Subunit (2)	1636	444 (27.1)	*1*
Adjuvanted (1)	526	114 (21.7)	0.74 (0.59–0.94)
Split (3)	660	138 (20.9)	0.71 (0.57–0.88)
Intradermal (1)	394	149 (38.1)	1.65 (1.31–2.08)
Age^c^			
≤ 14	651	208 (32.0)	1.98 (1.46–2.68)
15–25	142	70 (49.3)	4.10 (2.71–6.22)
26–65	1174	310 (26.4)	1.51 (1.14–2.02)
66–79	854	183 (21.4)	1.15 (0.85–1.56)
≥ 80	381	73 (19.2)	*1*
Sex			
Males	1614	373 (23.1)	*1*
Females	1599	472 (29.5)	1.39 (1.19–1.63)
Seasonal vaccination, 2014–2015^d^			
Yes	1958	490 (25.0)	*1*
No	805	241 (29.9)	1.28 (1.07–1.54)
High risk condition ^e^			
No	454	134 (29.5)	*1*
Yes	2253	581 (25.8)	0.83 (0.66–1.04)
Total	3213	845 (26.3)	

^a^ *vaccinated with one dose of flu vaccine*

^b^ *number of different products in brackets*

^c^ *Age not available for 11 subjects*

^d^ *Seasonal vaccination for 2014–2015 not available for 542 subjects*

^e^*Information on high risk condition not available for 506 subjects. High risk conditions include older people, pregnant women and people with chronic health conditions; these are the target for the influenza vaccination programme in Italy.* [8]

*OR odds ratio, CI confidence interval*

**Table 2:** Distribution of events within 7 days of vaccination by type of vaccine

Events	Number of vaccinated subjects^a,b^				
	Subunit	Split	Adjuvanted	Intradermal	Total
Local Symptoms					
Local pain	242	97	67	40	446
Local redness	81	22	13	106	222
Local swelling	93	26	15	75	209
Local Induration	84	19	14	57	174
Systemic Symptoms					
Malaise	64	15	23	20	122
Fever ≥37.5 °C	48	12	8	7	75
Headache	53	6	7	6	72
Arthralgia	20	5	10	7	42
Vomiting and nausea	20	6	4	6	36
Myalgia	14	15	6	3	28
Loss of appetite	15	6	1	3	25
Generalized itching	11	1	1	8	21
Irritability	13	0	0	0	13
Dyspnoea	5	2	0	3	10
Conjunctivitis	2	1	3	1	7
Rash	3	0	0	2	5
Persistent, inconsolable crying lasting ≥3 h	1	0	0	0	1
Urticaria	0	0	0	0	1
Others	44	4	10	25	83

^a^ *Vaccinated with one dose of flu vaccine*

^*b*^ *Total events may not equal the sum of individual symptoms reported, as vaccinated subjects were allowed to report multiple symptoms*

Younger ages (OR 1.98; 95% CI 1.46–2.68), females (OR 1.39; 95% CI 1.19–1.63) and individuals not vaccinated against influenza in the previous season (OR 1.28; 95% CI 1.07–1.54) reported significantly higher rates of events within 7 days of vaccination (Table 1).

In the multivariate logistic regression model age, sex and type of vaccine were associated with the occurrence of any event within 7 day of vaccination (Table 3). Assuming the subunit vaccines as reference category, the adjusted ORs were higher for intradermal vaccines (OR 3.53; 95% CI 2.58–4.84) and adjuvanted vaccines (OR 1.81; 95% CI 1.31–2.49). The multivariate analysis confirmed the highest rate of AEFIs occurred in younger vaccinated individuals (age ≤ 14 and 15–25 years) compared to those older than 75 years (OR 3.80; 95% CI 2.55–5.67 and OR 3.26; 95% IC 1.77–6.00, respectively) and in females compared to males (OR 1.51; 95% CI 1.27–1.80) (Table 3).

**Table 3:** Logistic regression analysis of variables associated with any event within 7 days of vaccination among 3213 subjects

	Adjusted OR (95% CI)
Age	
≤ 14	3.80 (2.55–5.67)
15–25	3.26 (1.77–6.00)
26–65	2.45 (0.90–1.72)
66–79	1.51 (1.17–1.80)
≥ 80	1
Sex	
Males	1
Females	1.51 (1.27–1.80)
Type of vaccines	
Subunit	1
Adjuvanted	1.81 (1.31–2.49)
Split	0.71 (0.53–0.96)
Intradermal	3.53 (2.58–4.84)

*Variables considered in the multivariate model are age at vaccination, sex and type of vaccines administered*

*OR odds ratio, CI confidence interval*

Among the 854 vaccinated subjects experiencing at least one AEFI within 7 days of vaccination, 829 subjects (97%) were contacted during the follow-up period at 60 days; 4 subjects reported that events were not fully resolved, while 25 subjects (2.9%) reported new AEFIs, most of them respiratory conditions.

## Discussion

Surveillance of adverse events following immunization is an important component of any national immunization programme because it is critical to assessing the safety of vaccines and to detecting potentially rare and severe adverse events and responding in a timely manner. This is even truer regarding the influenza vaccine because it is reformulated each year and annual revaccination is recommended. As available seasonal influenza vaccines safety profiles may vary between types and products and from year to year, active surveillance is crucial to monitor the possibility of adverse events occurring following manufacturing changes, contamination of batches or through the introduction of new pandemic influenza strains.

As seasonal influenza vaccines present several specific challenges for pharmacovigilance, more emphasis should be placed on the post-licensure monitoring of the benefit/risk of influenza vaccines, including a request for continuous monitoring of efficacy and enhanced safety surveillance that needs to be tailored according to where it is used.

This study aimed at assessing the safety of flu vaccines in the 2015–2016 season, through a protocol for active enhanced surveillance of AEFI. All individuals who approached the vaccine centres were invited to participate in the study. More than 3200 subjects, identified by the participating regions, were enrolled. However, this study has an important limitation: the adherence of vaccinated individuals was less than expected; therefore, it did not reach sufficient power to assess rarer AEs. In fact, the total number of recruited subjects is a very small proportion of the more than 3 million of vaccine doses administered in the participating regions during this season. This suggests that more efforts are needed in order to obtain sufficient numbers to identify rare events and to make comparisons between different vaccine brands or specific age groups. In the majority of cases, potential participants did not respond to recruitment efforts because of a general lack of interest and, for some, because of difficulties in communicating the information required during the consent process and in overcoming general resistance to participation in research studies. To encourage patient interest, approaching them one-on-one in their vaccine centre to inform about them about the project and developing additional strategies for follow up is crucial. Moreover, a variety of strategies to make potential participants aware of the study, including sending letters from the patient’s GPs and distributing flyers on influenza vaccination, could be useful. Furthermore, recently in Italy, there is a widespread skepticism and great hesitancy about vaccination, most of which is due to uncertainties regarding the safety profile of influenza vaccines [18].

Some variability by region regarding the number of those vaccinated was observed: this variation may depend on several factors, such as the size of the resident population, the vaccine coverage and, mainly, on the number and type of the vaccine centres participating to the study. In Italy, vaccine coverage in the general population (not just in all the target groups) in the 2015–2016 season was equal to 13.9%, and coverage in the participating regions ranged from 13.6 to 16.1%.

Our study aimed to actively follow-up defined cohorts of children, adults and the elderly at 7 days after immunization for specific AEFIs. The observed frequency of AEFIs that occurred within 7 days of influenza vaccination (26%), corresponded to other similar surveys conducted on elderly or paediatric populations [10–13], and it was consistent with the safety data of the vaccines [19]. These events were all mild, except for two deaths after administration of the influenza vaccine in subjects with underlying chronic conditions; clinicians excluded the correlation with the vaccination, according to the WHO criteria for AEFI [20]. Our study assessed the slightly higher risk for adjuvant and intradermal vaccines compared to the subunit and split (similar risks) vaccines, in line with Product Data Sheets and other comparison studies [21].

Stratified analysis showed variability in the frequency of mild events by type of vaccine and characteristics of the vaccinated individuals. The follow-up at 60 days of the 854 vaccinated individuals who reported at least one AEFI revealed a low occurrence of new symptoms (3%), and these were mainly respiratory and gastrointestinal symptoms. In 4 cases, however, the symptoms that occurred within the first 7 days continued to persist.

The study relied on two different methods to collect events recorded in the vaccine diary: the first method relied on a telephone interview with a vaccine centre operator and the second method directly involved the vaccinated person (or the parent, in the case of child) on a web platform. The web platform was tested at ISS and was shown to perform well, be easy to use and to provide good compliance; but, in this surveillance, fewer than 2% of the vaccinees used the web platform for data entry. This may be due to the lack of familiarity with informatics tools in those over the age 65 years, a group that made up 40% of the study population; the time was lacking to explain in more in detail the data entry system of health workers during the vaccine sessions. Although the additional information required during the vaccination was limited, we observed 14% of the data was missing, mainly concerning previous vaccinations, previous allergies, chronic illnesses and risk conditions. All this information is routinely gathered during the vaccination session but they are collected on paper and not always included in the IT (Information technology) systems available at vaccination centres. Therefore, automatic information collection systems should be adopted in this type of study to reduce operator workload and improve the quality of collected data. Otherwise, automated SMS-based reporting can facilitate sustainable, near real-time monitoring of adverse reactions and contribute to early identification of potential vaccine safety issues [22, 23]. Active surveillance of AEFI using SMS potentially permits more rapid identification of emerging safety signals. Also, compared with voice telephone interviews, data collection by SMS results in significantly improved response rates and timeliness of vaccine safety data [24].

However, our enhanced surveillance ensured to rapidly detect changes in the frequency and/or severity of expected reactogenicity that may indicate a potential for more serious risks as exposure to the vaccine increases. Moreover, it was able to generate the results during the entire vaccination campaign (October–December), as those vaccinated were enrolled and follow-up data was gathered 7 days after immunization.

## Conclusions

In conclusion, our data, even though they refer to a limited number of vaccinated subjects, are consistent with the safety of influenza vaccines in Italy during the 2015–2016 season for the most common adverse events. This could make a positive contribution towards improving the capability of public health systems to respond to demands concerning the safety surveillance system, even when the EMA guideline requirements were not closely met.

Mere registration and passive surveillance system reporting of possible adverse reactions are not enough to sustain confidence in the safety of the vaccines [25]. Active monitoring and systematic studies are important to test generated signals and hypotheses and to intensify awareness of the public and professionals with regard to the safety of vaccines [26].

To obtain enough power for comparative safety analyses, we are planning for next year’s season large cohort studies to identify long-term and rare adverse events through record linkage of different health databases. Alternatively, other approaches requiring only cases, some of which may use self-controlled techniques, can be applied [27–29].

## Additional file

Additional file 1: Case report forms of the study. Case report forms to collect information during vaccine administration, at 7 and 60 days after vaccine administration. (PDF 425 kb)

## Abbreviations

AEFI: Adverse events following immunization
CI: Confidence interval
CRF: Case report form
ED: Emerging department
EMA: European Medicines Agency
GP: General practitioners
ISS: Italian National Institute of Health
LHU: Local health units
OR: Odds ratio
SAE: Serious adverse events
SMS: Short message service
