# Supplementary material for: Active surveillance for safety monitoring of seasonal influenza vaccines in Italy, 2015/2016 season
Source: BMC Public Health. 2018 Dec 22;18:1401. doi: 10.1186/s12889-018-6260-5 (PMC6303938; doi:10.1186/s12889-018-6260-5)
Supplement: Supplementary file 1 — Case report forms of the study. Case report forms to collect information during vaccine administration, at 7 and 60 days after vaccine administration. (PDF 425 kb) [file 12889_2018_6260_MOESM1_ESM.pdf]

Sveva
SURVEILLANCE OF EVENTS FOLLOWING SEASONAL INFLUENZA VACCINATION
2015-2016 SEASON

SECTION 1: GENERAL INFORMATION

Region ______________________ **Centre** ______________ **ID |__|__|__|__|__|**

Form completed by ____**Date**___________________________

DEMOGRAPHIC CHARACTERISTICS

Surname____ First name ___________________

Fiscal Code |__|__|__| |__|__|__| |__|__|__|__|__| |__|__|__|__| |__| Gender ❑ M ❑ F

Date of birth |_|_| |_|_| |_|_|_|_| Place of birth ____

Nationality ❑ Italian ❑ Other (please specify) ____

Contact Nr ____________________________________ Preferred time to be contacted _________________________

Contact Nr of relatives/neighbors ____

If minor (under the age of 18 y):

Surname and first name of a parent:

____

____

Contact Nr ____________________________________ Preferred time to be contacted _________________________

PERSONAL INFORMATION OF THE VACCINEE

Relevant chronic diseases ❑ Yes ❑ No
If yes, please specify ____

Allergies ❑ Yes ❑ No
If yes, please specify ____

Use of drugs (within the last 3 months) ❑ Yes ❑ No
If yes, please specify ____

Other vaccination (within the last 2 weeks) ❑ Yes ❑ No
If yes, please specify date of administration and type of vaccine ____

Flu vaccination (previous season) ❑ Yes ❑ No

Was flu vaccination recommended to you because you are listed in a risk category? ❑ Yes ❑ No

SECTION 2: VACCINE ADMINISTRATION

ID |__|__|__|__|__|

Form completed by ____Date___________________________

Administration of the first dose

Contraindications to vaccination Yes ❑ No ❑

Date and route of vaccination |_|_| |_|_| |_|_|_|_| ❑ deltoid ❑ thigh ❑ gluteus

Vaccine brand: ____

Lot # ____ Expiration date |_|_| |_|_| |_|_|_|_|

Administered by ____

First dose diary received Yes ❑ No ❑

Password delivered Yes ❑ No ❑

Administration of the second dose

Contraindications to vaccination Yes ❑ No ❑

Date and route of vaccination |_|_| |_|_| |_|_|_|_| ❑ deltoid ❑ thig ❑ gluteus

Vaccine brand: ____

Lot # ____ Expiration date |_|_| |_|_| |_|_|_|_|

Administered by ____

Second dose diary received Yes ❑ No ❑

Password delivered Yes ❑ No ❑

SECTION 3: FOLLOW-UP AFTER 7 DAYS

ID |__|__|__|__|__|

Form completed by ____**Date**___________________________

Vaccinee contacted? Yes ❑ No ❑

If vaccinee is lost to follow up, please specify the reason ____

____

If minor (under the age of 18 y): ❑ Mother ❑ Father ❑ Other (please specify ___________________)

Administration ❑ 1° Dose ❑ 2° Dose

Vaccine brand ________________________ Date of administration |_|_| |_|_| |_|_|_|_|

Health status of vaccinee ____

Diary information

No events ❑

					Day from administration					
**YES**	**NO**	**Events**			**0**	**1**	**2**	**3**	**4**	**5**	**6**
❑	❑	Local redness	small ❑	large❑	❑	❑	❑	❑	❑	❑	❑
❑	❑	Local swelling	small ❑	large❑	❑	❑	❑	❑	❑	❑	❑
❑	❑	Local induration			❑	❑	❑	❑	❑	❑	❑
❑	❑	Local pain			❑	❑	❑	❑	❑	❑	❑
❑	❑	Fever≥37.5°C	body temperature (highest) |__|__|,|__|		❑	❑	❑	❑	❑	❑	❑
❑	❑	Loss of appetite			❑	❑	❑	❑	❑	❑	❑
❑	❑	Febrile seizures			❑	❑	❑	❑	❑	❑	❑
❑	❑	Headache			❑	❑	❑	❑	❑	❑	❑
❑	❑	Vomiting and nausea			❑	❑	❑	❑	❑	❑	❑
❑	❑	Malaise			❑	❑	❑	❑	❑	❑	❑
❑	❑	Arthralgia			❑	❑	❑	❑	❑	❑	❑
❑	❑	Myalgia			❑	❑	❑	❑	❑	❑	❑
❑	❑	Dyspnoea, asthma, bronchospasm			❑	❑	❑	❑	❑	❑	❑
❑	❑	Rash			❑	❑	❑	❑	❑	❑	❑
❑	❑	Generalized itching			❑	❑	❑	❑	❑	❑	❑
❑	❑	Conjunctivitis			❑	❑	❑	❑	❑	❑	❑
❑	❑	Urticaria			❑	❑	❑	❑	❑	❑	❑
❑	❑	Irritability (*only for children*)			❑	❑	❑	❑	❑	❑	❑
❑	❑	Persistent, inconsolable crying (*only for children*)	minute/hours |__|__|		❑	❑	❑	❑	❑	❑	❑
❑	❑	Other, please specify ______________________________			❑	❑	❑	❑	❑	❑	❑

NOTE ____

____

____

____

If the vaccinee reported at least one event, please specify:

Drug use for treatment of the event No ❑ Yes ❑

If yes, please specify what and when____

____

____

Medical visit No ❑ Yes ❑

If yes, please specify At home ❑ Ambulatory ❑ Hospital ❑ Emergency department ❑

Please specify the reason of the visit: ____

____

Hospitalization No ❑ Yes ❑

If yes, please specify Admission date |__|__| |__|__| |__|__| Length of hospital stay (days) |__|__|

Please specify the diagnosis of the hospital admission: _______________________________

____

Please specify the diagnosis of the hospital discharge: _______________________________

____

Event categories: Not serious ❑ Serious ❑ Not assessable ❑

SECTION 4: FOLLOW-UP AFTER 60 DAYS *(only for individuals with at least one event during the 7 day follow-up)*

ID |__|__|__|__|__|

Form completed by ____Date___________________________

Vaccinee contacted? Yes ❑ No ❑

If vaccinee is lost to follow up, please specify the reason ____

____

If minor (under the age of 18 y): ❑ Mother ❑ Father ❑ Other (please specify ____________________)

Administration ❑ 1° Dose ❑ 2° Dose

Vaccine brand _________________________ Date of administration |_|_| |_|_| |_|_|_|_|

Health status after 60 days from the onset of the event

General conditions Recovered without sequelae ❑ Not yet recovered ❑

Recovered with sequelae ❑ (Please specify _________________________)

Any other events within 60 days from the onset of the event Yes ❑ No ❑

If yes, please specify:

1. Date of onset of the event |_|_| |_|_| |_|_|_|_| Duration (days) |_|_|_|

   Brief description of the event ____

   ____

   ____

2. Date of onset of the event |_|_| |_|_| |_|_|_|_| Duration (days) |_|_|_|

   Brief description of the event ____

   ____

   ____

3. Date of onset of the event |_|_| |_|_| |_|_|_|_| Duration (days) |_|_|_|

   Brief description of the event ____

   ____

   ____

If the vaccinee reported at least one event, please specify:

Drug use for treatment of event No ❑ Yes ❑

If yes, please specify what and when ____

____

____

Medical visit No ❑ Yes ❑

If yes, please specify At home ❑ Ambulatory ❑ Hospital ❑ Emergency department ❑

Please specify the reason of the visit: ____

____

Hospitalization No ❑ Yes ❑

If yes, please specify Admission date |_|_| |_|_| |_|_| length of hospital stay (days) |_|_|

Please specify the diagnosis of the hospital admission: ____

____

Please specify the diagnosis of the hospital discharge: ____

____
